# Molecular Mechanism Investigation on Monomer Kaempferol of the Traditional Medicine Dingqing Tablet in Promoting Apoptosis of Acute Myeloid Leukemia HL-60 Cells

**DOI:** 10.1155/2022/8383315

**Published:** 2022-02-24

**Authors:** Dandan Zheng, Yongming Zhou, Yong Liu, Lihai Ma, Lingzhan Meng

**Affiliations:** ^1^Department of Oncology, Chongqing Hospital of Traditional Chinese Medicine, Chongqing, China; ^2^Department of Hematology, Yueyang Hospital of Integrated Traditional Chinese and Western Medicine, Shanghai University of Traditional Chinese Medicine, Shanghai, China

## Abstract

The traditional medicine Dingqing Tablet produces effective efficacy in treating acute myeloid leukemia, but its specific mechanism remains to be investigated. Dingqing Tablet consists of Codonopsis, Indigo Naturalis, Cortex Moutan, Radix Notoginseng, Citrus Reticulata, and Eolite. The active components of Dingqing Tablets were screened by the TCMSP database. Meanwhile, the SwissTargetPrediction database was utilized to predict the corresponding targets. Relevant disease targets of acute myeloid leukemia were obtained from GeneCards. The obtained targets of Dingqing Tablets and genes of acute myeloid leukemia were used, and the overlapped genes were presented in the Venn diagram. A drug-component-target network was constructed via Cytoscape 3.6.0 software. Molecular docking methodology was also used with AutoDock Vina 1.1.2. Furthermore, the effects of kaempferol on the proliferation and apoptosis of HL-60 cells were identified using 3-(4,5)-dimethylthiahiazo(-z-y1)-3,5-di-phenytetrazoliumromide (MTT), 5-Ethynyl-2′-deoxyuridine (EDU), flow cytometry, and TdT-mediated dUTP nick-end labeling (TUNEL) assays. The combination of kaempferol and AKT1 was verified using an immunoprecipitation (IP) experiment and the effects of Kaempferol on HL-60 cell apoptosis by western blot (WB) and qPCR. The key component kaempferol and the core target gene AKT1 were sorted out using a drug-component target network diagram. Molecular docking results revealed that the binding energy between kaempferol and AKT1 was lower than -5 kcal/mol. MTT and EDU assays indicated that kaempferol markedly inhibited the proliferation of HL-60 cells. Flow cytometry and TUNEL assays suggested that kaempferol substantially promoted HL-60 cell apoptosis. IP assay results testified that kaempferol could bind to AKT1, thereby reducing the level of P-AKT and promoting HL-60 cell apoptosis. The monomer kaempferol of Dingqing Tablet could promote apoptosis of HL-60 cells, and the mechanism might correlate with the combination of kaempferol and AKT1, reducing the level of P-AKT and promoting the expression of the apoptotic signaling pathway.

## 1. Introduction

Acute myeloid leukemia (AML) is recognized as a highly heterogeneous disorder and the most common acute leukemia developed in adults [1]. The American Cancer Society's 2021 estimate of leukemia in the United States is that there will be 20,240 new cases of AML, mostly adults, and about 11,400 deaths from acute myeloid leukemia, almost all of which occur in adults. AML is one of the most common types of leukemia in adults. AML is fairly rare overall, accounting for only about 1% of all cancers [2]. It is characterized by an abnormal proliferation of leukemia cells in the bone marrow. The clinical manifestations are anemia, hemorrhage, infection and pyrexia, organ infiltration, and metabolic abnormalities [3]. Currently, chemotherapy and stem cell transplantation are widely applied in the treatment of AML [4]. However, the prevalence of chemotherapy resistance and relapse remains a challenge for AML patients. Meanwhile, the five-year survival rate is still very low [5]. Further and deeper investigations have found that AML pathogenesis is complicated with multiple genes, multiple factors, and even multiple molecules involved [6, 7]. As AML patients develop general conditions of chemotherapy resistance, recurrence, and low survival rates, there is an urgent need to explore new biomarkers applied in diagnosing the occurrence, development, and prognosis of AML, as well as to discover novel therapeutic targets.

Dingqing Tablet serves as effective preparation for the treatment of leukemia. It has been developed by the team led by Professor Huang Zhenqiao from Yueyang Hospital of Integrated Traditional Chinese and Western Medicine, Shanghai University of Traditional Chinese Medicine, under the principles of Chinese medicine. This prescription consists of Codonopsis, Indigo Naturalis, Cortex Moutan, Radix Notoginseng, Citrus Reticulata, and Eolite [8]. Kaempferol is a main component of Cortex Moutan [9]. Kaempferol is also known as kaempferide with the chemical name of 3,5, 7-trihydroxy-2-(4-hydroxyphenyl)-4h-1-benzopyran 4-ketone, which belongs to the flavonol compounds [10]. This flavonoid compound is extensively present in edible plants and traditional natural medicines. Numerous preclinical studies have revealed that kaempferol and kaempferitrin exhibit extensive biological activities, including anticancer, anti-inflammation, antimicrobe, antioxidant, protection of both cardiovascular, and neuron as well as antidiabetes [11–13].

As a major intracellular signal transduction pathway [14], the PI3K-Akt signaling pathway is essential in promoting cell proliferation and inhibiting apoptosis in the body by influencing the activation of multiple downstream effector molecules [15]. Akt serves as a serine/threonine protein kinase (molecular weight 57 kD) [16]. The Bcl-2 family includes homodimers and heterodimers of which the formation and balance are of great significance to cell survival or apoptosis [17]. p-Akt promotes the phosphorylate of the Ser136/Ser112 residues of Bad, while Bcl-2 or Bcl-xl inhibited the phosphorylate of Bad [18]. The PI3K-Akt pathway activation enables phosphorylate the Ser184 residue of Bax, allowing Bax to stay in the cytoplasm, thereby promoting the formation of heterodimers and inhibiting cell apoptosis [18].

This research predicted the active ingredients and relevant targets of Dingqing Tablet employing a network pharmacology technique. The overlapped targets with disease target genes were subsequently obtained. Followed by core targets identification, we also conducted GO and KEGG pathway analysis and screened out the component kaempferol in Cortex Moutan. Molecular docking was subsequently conducted with the core target AKT1. Moreover, flow cytometry and TUNEL assays testified that kaempferol promoted apoptosis of HL-60 cells and determined that kaempferol and AKT could bind to each other and reduce the phosphorylation of AKT.

## 2. Materials and Methods

### 2.1. Cell Culture and Drug Treatment

Human leukemia cell line HL60 was purchased from American Type Culture Collection (ATCC). Culture medium utilized RPMI 1640 containing 10% inactivated fetal bovine serum, streptomycin, penicillin (each 100 U/mL), NaHCO_3_ (2 g/L), and HEPES (2.4 g/L) at PH 7.2–7.4, and incubation was performed at 37°C constant temperature and 5% CO_2_. Cells at the logarithmic growth phase were collected for further experiments, which were treated using kaempferol (25, 50, and 100 *μ*M), and the positive control was treated using all-trans retinoic acid (ATRA) (10 *μ*M).

### 2.2. Network Pharmacological Analysis

Through the network pharmacology approach, principal components and action targets of Dingqing Tablets were predicted. Firstly, the active ingredients of Dingqing Tablets (Codonopsis, Indigo Naturalis, Cortex Moutan, Radix Notoginseng, Citrus Reticulata, and Eolite) were retrieved from the TCMSP database [19] (https://tcmspw.com/tcmsp.php), and their corresponding targets were predicted using the SwissTargetPrediction website [20]. Human AML-related genes were sorted out from the gene disease database [21], and then a PPI protein interaction network diagram was constructed using String [22], and a drug-component-target network diagram was plotted using Cytoscape 3.6.0. A Cytoscape plugin ClueGO was used for GO and KEGG pathway analysis on the 92 targets, and visualization of the enrichment analysis results was processed [23].

### 2.3. Molecular Docking

The 3D structure of kaempferol was exported from TCMSP (https://tcmspw.com/tcmsp.php), and AKT 3D structure was retrieved from the Protein Data Bank [24] (http://www.rcsb.org/pdb). The receptor protein was added with hydrogen and calculated charge using AutoDock 4.2.6 software [25], docked with a ligand using AutoDock Vina 1.1.2 software. The docking results and the scores of binding energy were obtained. Those parts with the best binding energy were utilized for plotting. The 3D images plotted using PyMOL [26] presented the interaction between the receptor protein and the ligand.

### 2.4. MTT Assay

HL-60 cells were inoculated into a 96-well plate and followed by 6 d incubation. After being supplemented with 20 *μ*L MTT solution [27] in the wells, cell incubation was conducted, lasting for 4 h until being terminated. To each well, 150 *μ*L DMSO was added and shaken for 10 min [28]. The light absorption of each hole was measured on an enzyme-linked immunosorbent monitor at the wavelength of 490 nm for colorimetry. Cell growth curves were recorded and plotted [29].

### 2.5. EDU Assay

The HL-60 cells were planted to a 24-well plate. In light of the instructions of the EdU kit, a 2x EdU reaction solution was prepared and added to the 24-well plate. The cells were incubated in the dark for 2 h after the supplement of the reaction solution, fixed with 4% paraformaldehyde at room temperature for 20 min, supplemented with 500 *μ*L 0.3% Triton X-100, and reacted at room temperature for 10 min. Then, PBS was employed to rinse the cells 3 times. AZIDE 555-Click reaction solution was freshly prepared, supplied 200 *μ*L to each well, and incubated 30 min at room temperature avoiding light. After the reaction was completed, the solution was removed and followed by PBS washing 3 times, the nuclei were subsequently counterstained using Hoechst for further immunofluorescence. Observe and take pictures under an inverted microscope. Observation and photograph were performed under an inverted microscope [30].

### 2.6. Flow Cytometry

The cells were evenly inoculated into a 6-well plate, digested using tyrisin after 48 h transfection, and collected after centrifugation. Following two cycles of washing with precooled PBS, the cells were added with 400 *μ*L of 1 × Annexin V binding solution and suspended at a concentration of about 1 × 10^6^ cells/mL. The solution was transferred to a flow cytometry tube, supplied with 5 *μ*L Annexin V-FITC staining solution and mixed well gently. Incubation was followed under 4°C for 15 min avoiding light, supplemented with 10 *μ*L PI staining solution again and gently mixed, incubated for 5 min at 4°C in the dark followed by immediate detection by flow cytometry [31].

### 2.7. TUNEL Assay

The cell slides processed in experiments were treated with paraformaldehyde for 15–30 min at room temperature, washed 3 times with PBS, added with blocking solution, and incubated at room temperature for 10 min. Rinsed again with PBS, the slides were added with membrane solution and incubated 30 min at room temperature. The mixture of TUNEL reaction was prepared and mixed with 50 *μ*L TdT + 450 *μ*L fluorescein-labeled dUTP solution. Following reaction at room temperature for about 30 min, a 50 *μ*L mixture was supplied for reaction in a wet chamber avoiding light at 37°C for 60 min. Following 3 cycles of washing with PBS, one PBS was dripped, and the apoptotic cells were observed using a fluorescence microscope (the excitation and detection wavelength were 450–500 nm and 515–565 nm, respectively) [32].

### 2.8. Coimmunoprecipitation Assay

Biotin-labeled kaempferol, the kit used EZ-Link™ Biotin-LC-Hydrazide (Thermo Scientific), and the procedures were followed following the operating instructions. Biotin-labeled kaempferol was inoculated into HL-60 cell suspension, cultured for 24 h, and centrifuged to collect the cells. Precooled RIPA Buffer was added, and the cells were collected for centrifugation at 14 000 g 15 min. Protein A agarose was prepared following two cycles of bead washing with PBS. To each 1 mL of total protein, 100 *μ*L Protein A agarose beads (50%) were added and centrifuged at 14 000 g for 15 min. Rabbit antibody was subsequently added, and the antigen-antibody mixture was slowly shaken at 4°C overnight, added with 100 *μ*L protein A agarose beads, and shaken slowly overnight at 4°C. Instantaneous centrifugation was performed at 14 000 rpm for 5 s before the collection of agarose bead-antigen-antibody complex and followed by electrophoresis [33].

### 2.9. WB Assay

The cells were harvested for lysis using IP lysis buffer. Following centrifugation at 4°C 13 000 rpm 20 min, the supernatant was obtained, and the protein was harvested. 10% SPS-PAGE was employed to separate the total protein, which was then transferred to PDVF, blocked with milk at room temperature for 1 h with subsequent TBST washing 3 times. Primary antibody was supplied and incubated overnight at 4°C, and second antibody was supplied the next day and incubated for 1 h for ECL coloration [34].

### 2.10. qPCR Assay

Extraction of total RNA was conducted employing Trizol reagent (Takara, Japan). The first reversely transcribed cDNA strand used the PrimeScript™ RT kit (Takara, Japan). When determining relative gene expression, the reaction system and procedures of qPCR referred to instructions of TBGreenPremixExTaqII (Takara, Japan), and the instrument was CFX96Real-Timesystem (Bio-Rad, USA). The 2^−∆∆CT^ algorithm was used to calculate the relative gene expression levels [34].

### 2.11. Data Analysis

The experimental data obtained were exhibited as mean ± standard deviation (SD). Student's *t*-test was performed for pairwise comparison and one-way analysis of variance (ANOVA) for multiple group comparison to assess the significance of statistical data using GraphPad Prism 7.0 software (LaJolla, CA, USA). *p* values less than 0.05 were regarded as the difference was statistically significant.

## 3. Results

### 3.1. Overlapped Targets and Network Visualization of Drugs and Diseases

**Figure 1:**
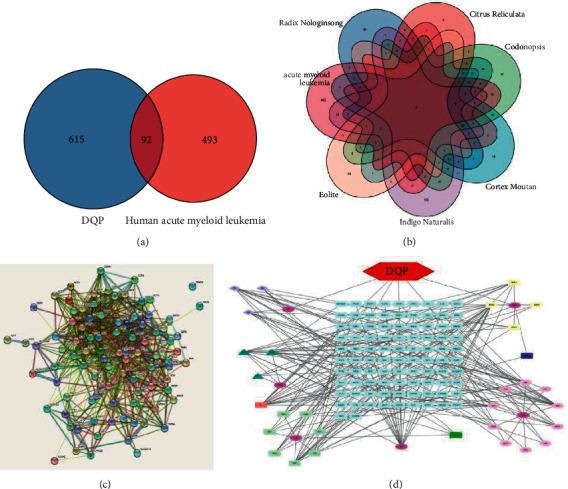
Analysis of the regulatory network of Dingqing tablets-key components-targets-acute myeloid leukemia based on network pharmacology. (a) Venn diagram of overlapped genes between Dingqing Tablet and human AML targets. (b) Venn diagrams of overlapped genes between Radix Notoginseng, Citrus Reticulata, Codonopsis, Cortex Moutan, Indigo Naturalis, and Eolite and human AML targets. (c) Protein-protein interaction analysis of 92 proteins. (d) Drug-active ingredient-target gene network diagram. DQP represented the compound drug Dingqing Tablet, and SSQ, CP, DS, MDP, QD, and XH, respectively, represented the six herbs containing in Dingqing Tablet: Radix Notoginseng, Citrus Reticulata, Codonopsis, Cortex Moutan, Indigo Naturalis, and Eolite.

To explore the actions of Dingqing Tablet on leukemia HL-60 cells, we initially determined the active ingredients and relevant targets of Dingqing Tablets. There were 707 targets sorted from Dingqing Tablet and 585 targets from human AML (Figure 1(a)). The overlapped genes of Radix Notoginseng, Citrus Reticulata, Codonopsis, Cortex Moutan, Indigo Naturalis, and Eolite and human AML were 37, 6, 97, 15, 102, and 58, respectively (Figure 1(b)). A PPI protein interaction network diagram was constructed through String (Figure 1(c)). The drugs, ingredients, and targets were visualized via Cytoscape 3.6, we found that MDP2 (kaempferol), DS6 (luteolin), XH (eolite), and E1 (quercetin) were the key ingredients in Dingqing Tablet, and AKT and STAT3 were the core target genes with top degrees (Figure 1(d)).

### 3.2. Molecular Docking

**Figure 2:**
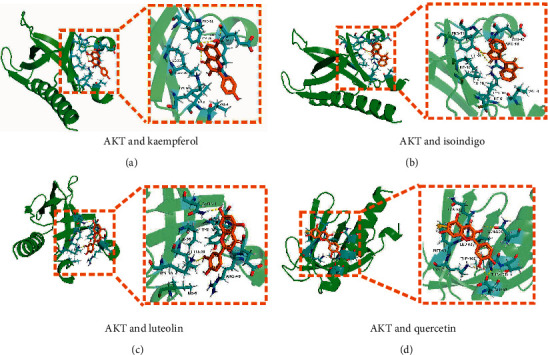
Molecular docking of the key components in Dingqing Tablet and the core target AKT1. (a) Molecular docking of AKT and kaempferol. (b) Molecular docking of AKT1 and isoindigo. (c) Molecular docking between AKT1 and luteolin. (d) Molecular docking between AKT1 and quercetin.

We selected the key ingredient kaempferol and the core target genes AKT1 (PDB ID: 1UNQ) and XDH (PDB ID: 5DN2) to conduct molecular docking with its corresponding chemical components. As shown in Figure 2, the binding energies of AKT1 and the main chemical components kaempferol (Figure 2(a)), isoindigo (Figure 2(b)), luteolin (Figure 2(c)), and quercetin (Figure 2(d)) were −5.16, −5.91, −5.37, and −5.45 kcal/mol, respectively. The binding between kaempferol and AKT1 exhibited that the amino acid residues Arg48 and Tyr38 generated hydrogen bond interactions, while the amino acid residues Pro51, Glu49, Ile36, Leu28, Lys30, Ile6, Val4, and kaempferol formed hydrophobic interactions.

### 3.3. GO and KEGG Pathway Enrichment Analysis

**Figure 3:**
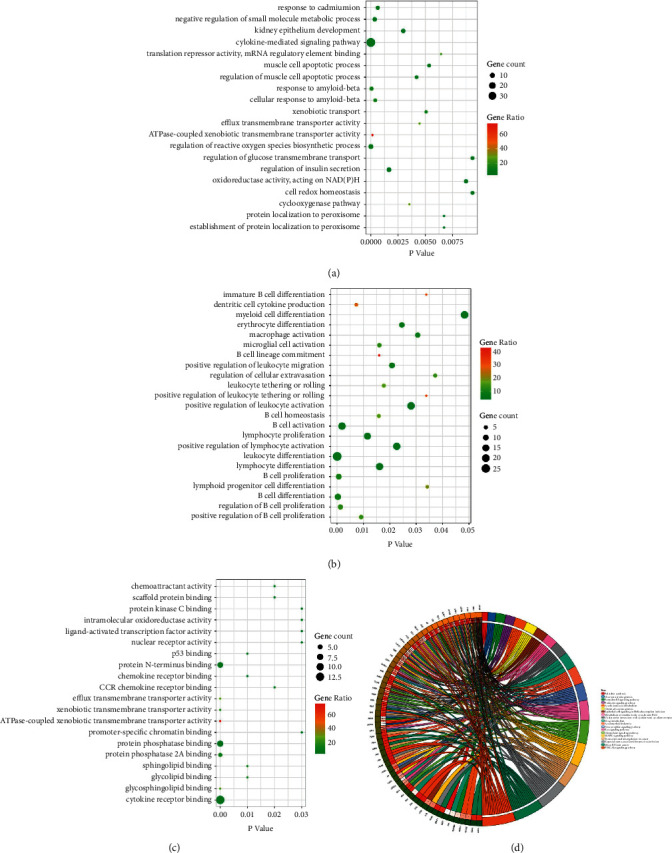
GO and KEGG pathway enrichment analysis of Dingqing Tablet in AML treatment (a, b, c) Bubble charts of biological process, immune response, the molecular function of overlapped genes using GO analysis. The *Y*-axis on the left represented entry names of GO analysis, and the *X*-axis represented *p* values. Big circles represented that more genes were aligned with the pathway, and darker colors represented a higher proportion of the aligned genes in the pathway. (d) Analysis results of KEGG pathway enrichment. The outermost circle on the right indicated entry names of the signaling pathways, and the left indicated genes. The inner circle on the left indicated the significance of *p* values of the pathways corresponding to the gene.

We had a further illustration of the target function of Dingqing Tablet and the role of potential targets in the signaling pathway and analyzed the 92 targets through GO and KEGG pathway analysis employing a Cytoscape plugin ClueGO. Meanwhile, visualization of the enrichment analysis results was processed. As shown in Figure 3, GO analysis indicated that the most prominent biological processes included negative regulation of small molecule metabolic process and translation repressor activity; and those included in cell components and molecular functions were ligand-activated transcription factor activity, mRNA regulatory element binding, myeloid cell differentiation, and nuclear receptor activity (Figure 3(a)). KEGG pathway analysis results revealed that the 92 potential targets of Dingqing Tablet in AML treatment were mainly correlated with the MAPK signaling pathway and PI3K-Akt signaling pathway (Figure 3(b)).

### 3.4. Monomer of Dingqing Tablet Inhibits the Proliferation of HL-60

**Figure 4:**
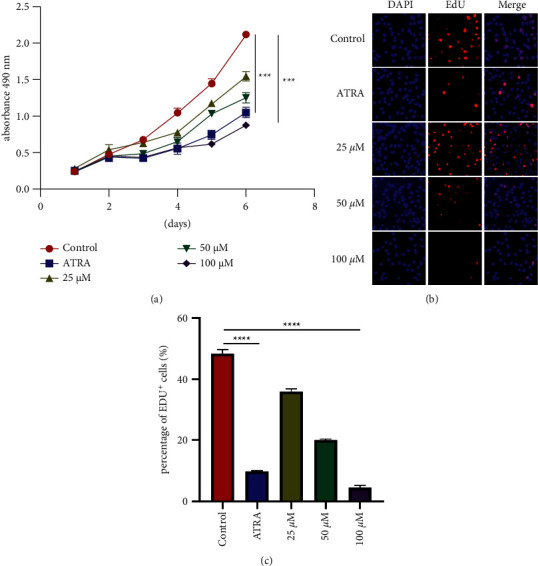
Kaempferol inhibits HL-60 cell proliferation. (a) HL-60 cell growth curves. HL-60 cells were treated with kaempferol (25 *μ*M, 50 *μ*M, and 100 *μ*M) and ATRA (10 *μ*M) for 72 h (b, c) Representative images (b) and quantification (c) were obtained via EdU incorporation assays. HL-60 cells were treated with kaempferol (25 *μ*M, 50 *μ*M, and 100 *μ*M) and ATRA (10 *μ*M) for 48 h. Data were represented as mean ± SD (*n* ≥ 3).  ^*∗*^*p*=0.05,  ^*∗∗*^*p*=0.01,  ^*∗∗∗*^*p*=0.001,  ^*∗∗∗∗*^*p*=0.0001 as determined using Student's *t*-test for pairwise comparison or one-way ANOVA, followed by Tukey's test for multiple group comparison.

To explore influences of Dingqing Tablet monomer kaempferol on the proliferation of HL-60, we divided HL-60 into a Control group, a Cisplatin group, a 25 *μ*M kaempferol group, a 50 *μ*M kaempferol group, and a 100 *μ*M kaempferol group. We detected the cell proliferation using MTT assays, and the results revealed that ATRA and 100 *μ*M kaempferol could markedly suppress the proliferation of HL-60 cells on day 4 (*p*=0.0213 and *p*=0.0306) (Figure 4(a)). Furthermore, the action of kaempferol on the proliferation ability of HL-60 cells was detected using EDU experiment, and the results revealed that 100 *μ*M kaempferol could markedly suppress the proliferation ability of HL-60 cells (Figures 4(b)–4(c)).

### 3.5. Monomer of Dingqing Tablet Promotes HL-60 Apoptosis

**Figure 5:**
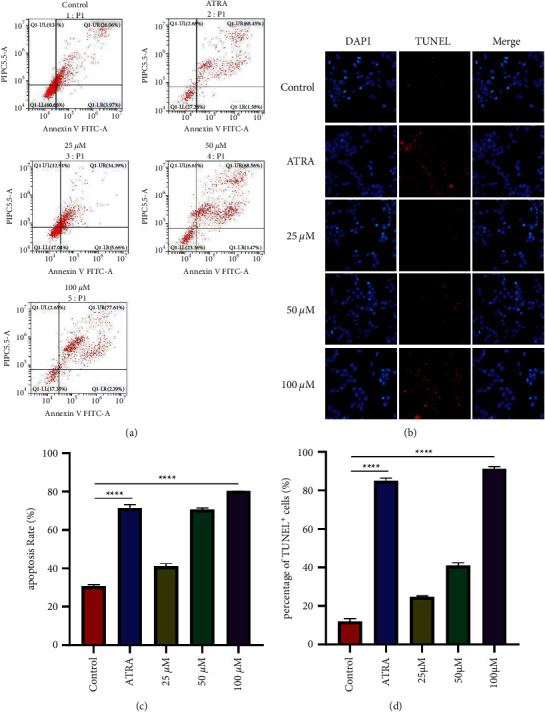
Kaempferol promotes apoptosis of HL-60 cells. (a) Flow cytometry analysis of apoptosis was performed following kaempferol treatment (25 *μ*M, 50 *μ*M, and 100 *μ*M) and ATRA (10 *μ*M). (b) TUNEL analysis of apoptosis following kaempferol treatment (25 *μ*M, 50 *μ*M, and 100 *μ*M) and ATRA (10 *μ*M). (c) Quantification of cell death detected in Figure 4(c). (d) Quantification of TUNEL+cell in Figure 4(b). Data obtained were expressed as mean ± SD (*n* ≥ 3).  ^*∗*^*p*=0.05,  ^*∗∗*^*p*=0.01,  ^*∗∗∗*^*p*=0.001,  ^*∗∗∗∗*^*p*=0.0001 based on Student's *t*-test (two groups) or one-way ANOVA and Tukey's test for more than two groups.

To clarify the effect of kaempferol on HL-60 apoptosis, we performed flow cytometry and TUNEL assays to determine whether kaempferol acted on HL-60 cell apoptosis. Flow cytometry results revealed that the 100 *μ*M kaempferol group substantially promoted apoptosis of HL-60 cells compared with the control group (Figures 5(a) and 5(c)). And TUNEL assays indicated that 100 *μ*M kaempferol remarkably elevated the percentage of TUNEL+ cells (%) compared with the control group (Figures 5(b) and 5(d)).

### 3.6. Kaempferol Dingqing Tablet Combined with AKT to Promote the AKT-Bcl2 Signaling Pathway Activation

**Figure 6:**
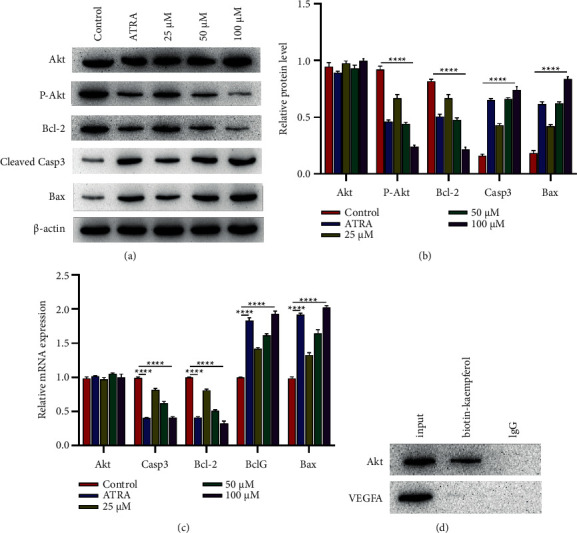
Kaempferol promotes the expression of apoptotic pathway. (a) Protein expressions of Akt, P-Akt, Bcl2, Cleaved Casp3, and Bax. HL-60 cell treatment presented as in Figure 4(a) and the protein isolation and analysis by western blotting. GAPDH expression served as an internal control. (b) TUNEL findings after kaempferol treatment (25 *μ*M, 50 *μ*M, and 100 *μ*M) and ATRA (10 *μ*M). (c) mRNA expression levels of the indicated genes were measured by qRT-PCR. (d) Coimmunoprecipitation assay shows AKT1-kaempferol interactions in HL-60 cells. Data were expressed as mean ± SD (*n* ≥ 3).  ^*∗*^*p*=0.05,  ^*∗∗*^*p*=0.01,  ^*∗∗∗*^*p*=0.001,  ^*∗∗∗∗*^*p*=0.0001 based on Student's *t*-test (two groups) or one-way ANOVA and Tukey's tests for more than two groups.

To explore whether kaempferol was correlated with the downstream apoptosis signaling pathway of AKT1, WB results indicated that kaempferol could increase the expression of apoptotic proteins by inhibiting P-AKT levels (Figures 6(a) and 6(b)). Meanwhile, kaempferol could markedly promote apoptosis of HL-60 detected by qPCR (Figure 6(c)). As verification of the relationship between kaempferol and AKT1 was required, we labeled kaempferol with biotin to observe whether it could bind to AKT1 or not. IP results revealed that kaempferol could combine with AKT1 (Figure 6(d)).

## 4. Discussion

AML is a malignant clonal disease originating from the hematopoietic system. The leukemia cells lose control in proliferation and develop differentiation dysfunction and apoptosis obstruction during cell cycles [35]. They can aggregate in the bone marrow and certain hematopoietic tissues in a great number, thereby inhibiting the normal hematopoietic function of bone marrow and infiltrating multiple organs [36]. As techniques in chemotherapy, specifically targeted therapy, and bone marrow transplantation advance constantly in recent years, the treatment effect has been continuously improved [36]. The overall disease remission rate of AML patients can reach 50% to 80%. However, approximately 65% of patients remain to undergo relapse within 3 to 5 years [37]. The overall survival rate of adults is only 24% to 28% [38].

Traditional Chinese medicine presents unique benefits in antitumor and immune function regulation of the body. In recent years, Chinese medicine has been commenced gradually in clinically treating malignant hematological diseases [39]. Despite the fact that Dingqing Tablet developed by the team led by Professor Huang Zhenqiao can effectively treat leukemia, its specific mechanism has not yet been elucidated. We screened the traditional Chinese medicine monomer kaempferol and AKT interaction using network pharmacology and molecular docking and verified its downstream apoptosis signaling pathway.

First, the 38 active ingredients of Dingqing Tablet and their corresponding 615 targets were identified. A total of 92 overlapped targets of Dingqing Tablet and AML targets were obtained subsequently. Second, the constructed PPI network diagram via the String platform revealed that proteins VEGFA, STAT3, AKT1, and TNF were located in the center of the network and interacted more frequently with other factors. The results suggested that AKT1 might be a key target for the AML treatment. The drugs, ingredients, and targets were visualized using the Cytoscape software. We found that kaempferol, luteolin, Eolite, and quercetin had the most connections, and they might be the key ingredients of Dingqing tablet. Next, we carried out molecular docking, and the binding energies between AKT1 and the main chemical components kaempferol, isoindigo, luteolin, and quercetin were −5.91, −5.16, −5.37, and −5.45 kcal/mol, respectively, of which kaempferol and AKT1 had the lowest binding energy. It was obvious that kaempferol was very likely to treat AML by acting on AKT1. Recently, a great number of studies have reported that kaempferol can promote tumor cell apoptosis by regulating the PI3K-AKT pathway [39–41].

Subsequently, the function of the targets of Dingqing Tablet was testified and whether the potential targets played a role in the signaling pathway was also assessed. The 92 targets were analyzed using GO and KEGG pathway analysis with the Cytoscape ClueGO software. GO analysis results demonstrated that the collected targets might affect the negative regulation of the small molecule metabolic process and translation repressor activity in the biological process. Through analyses of cell components and molecular functions, the previously described targets might influence mRNA regulatory element binding, myeloid cell differentiation, ligand-activated transcription factor activity, and nuclear receptor activity. KEGG pathway analysis results exhibited that the 92 potential targets of Dingqing Tablet in AML treatment were mainly correlated with both PI3K-Akt and MAPK signaling pathways, of which AKT served as a core protein of the PI3K-Akt signaling pathway. We hypothesized that kaempferol in Dingqing Tablet directly acted on AKT to treat AML by regulating the PI3K-Akt signaling pathway.

To clarify how the monomer of Dingqing Tablet kaempferol acted on the proliferation of HL-60, we conducted MTT experiments, which suggested that kaempferol could inhibit the proliferation of HL-60 cells. Furthermore, an EDU experiment was conducted to determine the effect of kaempferol on the proliferation ability of HL-60 cells, and consistent results were obtained. To explore the effect of kaempferol on HL-60 apoptosis, we performed flow cytometry and TUNEL experiments, which proved that kaempferol promoted HL-60 cell apoptosis.

To verify the intrarelationship between kaempferol and core targets AKT1 and VEGFA, we employed a biotin-labeled IP technique to observe the binding of kaempferol target proteins. IP results suggested that kaempferol could combine with AKT1. To explore whether kaempferol was linked to the AKT1 downstream apoptosis signaling pathway, WB and qPCR results demonstrated that kaempferol could elevate the expression of apoptotic proteins and reduce the expression of anti-apoptotic proteins by suppressing P-AKT levels. qPCR assays also proved that kaempferol could promote apoptosis of HL-60 cells. The previously described experiments demonstrated that kaempferol could bind to AKT1, thereby reducing the levels of P-AKT and promoting apoptosis of HL-60 cells.

Taken together, the main components and targets of Dingqing Tablet were predicted in a network pharmacological approach. Meanwhile, molecular docking was employed to determine the key components kaempferol and the core overlapped protein AKT1. The IP experiment demonstrated that kaempferol and AKT1 could interact with each other, and further findings indicated that kaempferol reduced the levels of P-AKT and promoted apoptosis of HL-60 by binding to AKT1.

## 5. Conclusion

The monomer kaempferol of Dingqing Tablet could promote apoptosis of HL-60 cells. The mechanism of Dingqing Tablet might correlate with the combination of kaempferol and AKT1, reducing the level of P-AKT and promoting the expression of the apoptotic signaling pathway.

## Data Availability

The data used to support the research were included within this manuscript.
